# Estimation of the Human Extrathoracic Deposition Fraction of Inhaled Particles Using a Polyurethane Foam Collection Substrate in an IOM Sampler

**DOI:** 10.3390/ijerph13030292

**Published:** 2016-03-07

**Authors:** Darrah K. Sleeth, Susan A. Balthaser, Scott Collingwood, Rodney R. Larson

**Affiliations:** 1 Rocky Mountain Center for Occupational & Environmental Health, Department of Family & Preventive Medicine, University of Utah, Salt Lake City, UT 84108, USA; sbalthaser@gmail.com (S.A.B.); rod.larson@hsc.utah.edu (R.R.L.); 2 Department of Pediatrics, University of Utah, Salt Lake City, UT 84108, USA; scott.collingwood@hsc.utah.edu

**Keywords:** extrathoracic deposition, polyurethane foam, IOM sampler, inhalable particles, oral passage, nasal passage

## Abstract

Extrathoracic deposition of inhaled particles (*i.e.*, in the head and throat) is an important exposure route for many hazardous materials. Current best practices for exposure assessment of aerosols in the workplace involve particle size selective sampling methods based on particle penetration into the human respiratory tract (*i.e.*, inhalable or respirable sampling). However, the International Organization for Standardization (ISO) has recently adopted particle deposition sampling conventions (ISO 13138), including conventions for extrathoracic (ET) deposition into the anterior nasal passage (ET_1_) and the posterior nasal and oral passages (ET_2_). For this study, polyurethane foam was used as a collection substrate inside an inhalable aerosol sampler to provide an estimate of extrathoracic particle deposition. Aerosols of fused aluminum oxide (five sizes, 4.9 µm–44.3 µm) were used as a test dust in a low speed (0.2 m/s) wind tunnel. Samplers were placed on a rotating mannequin inside the wind tunnel to simulate orientation-averaged personal sampling. Collection efficiency data for the foam insert matched well to the extrathoracic deposition convention for the particle sizes tested. The concept of using a foam insert to match a particle deposition sampling convention was explored in this study and shows promise for future use as a sampling device.

## 1. Introduction

The nose is the first significant human exposure route for many inhaled substances. Extrathoracic deposition (*i.e.*, in the head and throat, including the nasal and oral passages) is therefore an important exposure route for many hazardous materials. Nasal inhalation has been associated with adverse health effects including nasal cancer [1], nasal irritation [2], nasal septal perforation [3], allergic rhinitis [4], and sensitization [5]. Approximately one-third (31%–43%) of nasal cancer deaths in the U.S. are considered occupationally-related [6]. Formaldehyde (a known carcinogen [7]) is almost exclusively absorbed in the nose after inhalation [2]. Wood dust, nickel and hexavalent chromium are common exposures with potentially serious nasal effects, with an estimated 12 million U.S. workers exposed [6]. These numbers do not include other health outcomes related to substances that deposit in the extrathoracic region, such as systemic effects or occupational asthma. Altogether, these represent a significant health burden(s) to workers.

No personal air samplers for occupational or environmental exposure assessment currently provide a simple measurement of extrathoracic particulate deposition. There are commercially-available models of human throats that enable estimation of extrathoracic particle deposition, including the “Alberta Idealized Throat” often used for medical inhaler testing [8,9,10]. However, most existing personal sampling methods for airborne dust in workplaces measure either *"total dust"* (e.g., the 37-mm closed face cassette sampler) or respiratory tract particle *penetration* (*i.e*., what enters into the body, not necessarily what deposits there). Nasal exposures may result from two different mechanisms: either (1) particles impacting on the nasal mucosa during inhalation (typically large particles); or (2) particles depositing on exhalation (typically smaller particles). Currently available samplers do not accurately assess these exposures. For example, respirable cyclones, which remove larger particles, provide measures of particulates that would penetrate the deep lung. However, a large fraction of what is inhaled does not deposit in the lower respiratory tract (*i.e.*, the lungs) and thus may be misclassified in terms of its physiologically-relevant site of action. The existing sampling methods which measure penetration fractions thereby have the potential to provide inaccurate exposure data for either industrial hygiene surveys or occupational epidemiological studies. The development of samplers to measure deposition in the extrathoracic region is therefore of ongoing interest. An extrathoracic deposition sampler is needed to provide accurate exposure assessment data, which are vital to perform valid and reliable industrial hygiene and occupational epidemiological studies.

Recently, personal samplers have been developed that measure total particle deposition along the entire respiratory tract [11], regional particle deposition [12] and nanoparticle deposition in the lung [13]. An area sampler has also been designed to follow the International Committee for Radiological Protection (ICRP) model of particle deposition in the human body [14]. However, that sampler is only valid for particles up to 20 µm in size [15], so it does not cover the entire inhalable size range of particles (up to 100 µm) including those relevant to the extrathoracic airways. This study aimed to develop an air sampler that specifically (and uniquely) measures particle deposition in the extrathoracic region.

Due to the variability in human breathing, models of particle deposition in the human body, such as those published by ICRP [14], need to be simplified into “sampling conventions” so that targeted air sampling devices might be developed. Therefore, of primary importance to size-selective air sampler development is a metric (*i.e.*, a sampling convention or target specification) against which to compare such a device. The International Organization for Standardization (ISO) has recently adopted sampling conventions for particle deposition in different respiratory tract locations [16]. These are separated into extrathoracic (ET_1_ for the anterior nasal passages and ET_2_ for the posterior nasal passages and oral passages), tracheobronchial (BB and bb), and alveolar fractions, with different conventions for particles in the aerodynamic (>0.5 µm) and thermodynamic (<0.5 µm) size ranges.

The ISO conventions are averaged over different breathing conditions and for both males and females. This allows for the use of a single convention for each respiratory tract region, which is necessary for development of a sampling device that could be utilized in the field by industrial hygienists and other exposure scientists. As it relates to exposures in the extrathoracic region, these conventions are separated into ET_1_ (Equation (1)) and ET_2_ (Equation (2)), described for the aerodynamic size range (>0.5 µm) by the following cumulative lognormal functions, with *d_ae_* being the particle aerodynamic diameter (in µm):

(1) $$D_{aET_{1}}\left[d_{ae}\right]=0.325F\left[d_{ae},\left(d_{c},\sigma \right)\right]$$

Here the median cut diameter is given by $d_{c}\left(\mu m\right)=2.7$ and the standard deviation (variance) is given by σ=ln[2.5].

(2) $$D_{aET_{2}}\left[d_{ae}\right]=2.080\;D_{aET_{1}}\left[d_{ae}\right]$$

Note that ET_2_ (Equation (2)) is dependent on ET_1_ (Equation (1)). In fact, the total ET deposition efficiency (Equation (3)) can be obtained by replacing the constant “0.325” with “1.000,” which is then given simply by:

(3) $$D_{aET}\left[d_{ae}\right]=F\left[d_{ae},\left(d_{c},\sigma \right)\right]$$

where $d_{c}\left(\mu m\right)=2.7$ and σ=ln[2.5].

**Figure 1:**
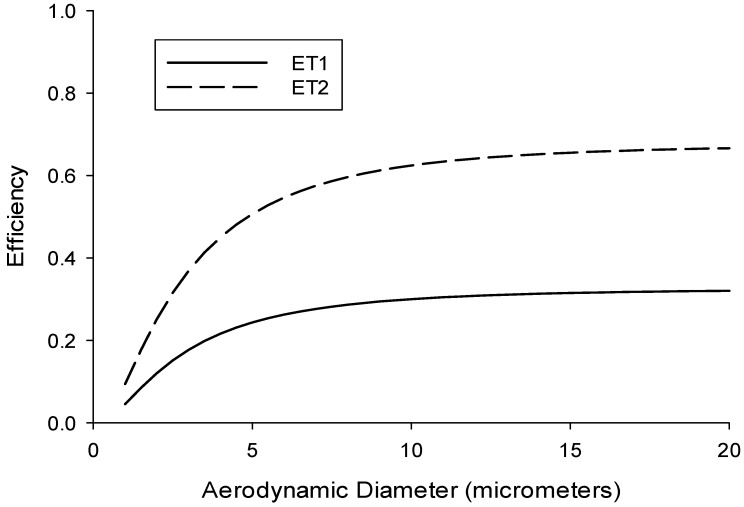
International Organization for Standardization (ISO) sampling conventions for extrathoracic (ET) deposition of particles in the human respiratory tract [16]. ET_1_ (solid line) represents deposition in the anterior nasal passages, ET_2_ (dashed line) represents deposition in the posterior nasal passages and oral passages.

**Figure 2:**
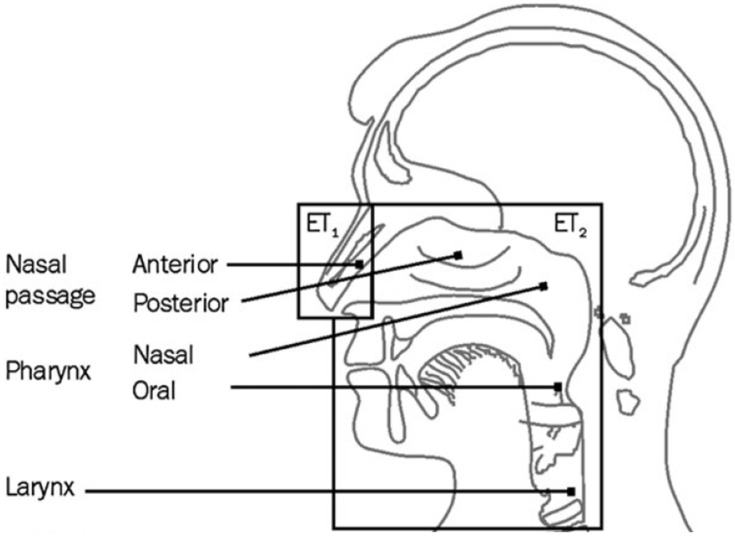
Diagram showing the various locations of potential extrathoracic particle deposition in humans [18].

This is, in fact, identical to the sum of ET_1_ and ET_2_. This function reflects the fact that at large *d_ae_* all aerosol particles that enter into the nose and/or mouth deposit in the extrathoracic region. Curves for ET_1_ and ET_2_ are shown in Figure 1, with the anatomical descriptions of these two conventions shown in Figure 2.

**Figure 3:**
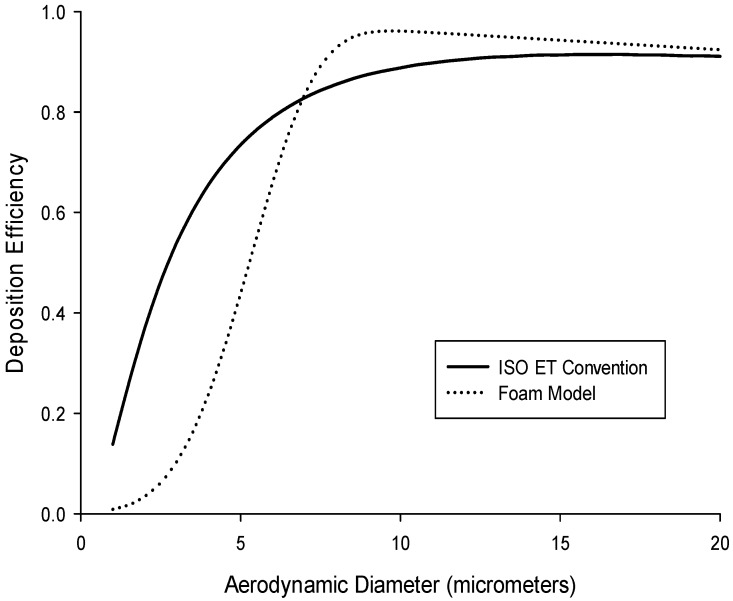
The Institute of Occupational Medicine (IOM) Multidust foam deposition efficiency modeled using Equation (6), for a sampler flow rate of 2 L/min (dotted line), compared to the extrathoracic (ET) convention (solid line) (Equation (4)), both normalized with the low-wind inhalability convention.

While the ultimate target specification is the extrathoracic deposition convention, it is also of interest to use a sampler that incorporates a target specification for the inhalable fraction to simplify sampler design for estimating deposition in the extrathoracic region. In other words, the conventions shown in Figure 1 do not account for the fact the particles can only deposit once they have been inhaled. This suggested “normalized” criterion can be seen in Figure 3 (solid line), which represents the ET convention multiplied by the low-velocity inhalability criterion [17]. From this, it is more obvious that above a certain particle size, all inhaled particles will deposit in the nose, and will not reach further into the respiratory tract. The advantage of this recommendation is that it would enable use of an air sampler that captures the inhalable fraction to be used as a baseline sampler for measuring extrathoracic particle deposition. This normalized ET deposition criterion, based on the total extrathoracic deposition efficiency (Equation (3)), is described by the following cumulative probability function (*F*):

(4) $$D_{ETnormalized}\left[d_{ae}\right]=I\left(d_{ae}\right)F\left[d_{ae},\left(d_{c},\sigma \right)\right]$$

where $d_{c}\left(\mu m\right)=2.7$ and σ=ln[2.5], and *I(d_ae_)* is the inhalable aerosol fraction as a function of aerodynamic particle size (in µm) for low wind speed environments [17] given by the following equation:

(5) $$I\left(d_{ae}\right)=1-0.0038d_{ae}$$

It should be mentioned that the inhalability criterion (Equation (5)) is a conservative estimate of what enters into the nose and/or mouth. In this way, the possibility exists that it may lead to an overestimate of what deposits solely in the nose. In practice, for example in a study on individual workers for which correlation to health outcomes is desired, conversion factors that account for the difference between oronasal breathers and mouth-only breathers are provided in an Annex of ISO.

Standard 13138. Those correction factors also account for differences between sitting, light exercise and heavy exercise. For the current study, however, these differences are not taken into account since individual estimates of dose are not of interest. The purpose of the current study was to design and test a simple sampler that would approximate the extrathoracic deposition of inhaled particles. By developing a sampler that measures the average extrathoracic deposition fraction, the relevant correction factors could ultimately be applied in practice, thereby taking into consideration individual worker characteristics. This would provide a useful tool for improved exposure assessment estimates, especially for substances with known extrathoracic health outcomes, such as wood dust and welding fume.

## 2. Materials and Methods

### 2.1. Sampler Design

Based on the idea that an inhalable aerosol sampler might provide the basis for a sampler that estimates extrathoracic particle deposition, the Institute of Occupational Medicine (IOM) inhalable sampler was investigated as a candidate sampler. That sampler was designed specifically to measure those particles that can penetrate into the nose and/or mouth during breathing [19]. It consists of a conductive plastic housing with a stainless steel cassette insert, which holds the sampling filter. Both the filter and any deposits inside the cassette itself are analyzed as part of the sample.

For the purposes of this study, polyurethane foam (PUF) was chosen as a collection substrate as it would select out only those particles that are likely to deposit in the extrathoracic region (*i.e.*, the larger particles). Polyurethane foam used as a particle separation device has been reported previously as being able to successfully collect the respirable or thoracic particulate fractions [20,21,22], including bioaerosols [23]. In fact, a PUF insert already commercially available for use in the IOM sampler separates out the respirable penetration fraction (IOM Multidust sampler, SKC Inc., Eighty-four, PA, USA). In this way, it provides pre-separation of non-respirable particles (which are collected by the foam) to enable the so-called IOM Multidust sampler to simultaneously measure inhalable (<100 µm) and respirable (<5 µm) particulate exposures. The foam, by itself, is not typically analyzed. This insert measures at 1.65 cm in diameter, 1.2 cm in thickness and with approximately 85 pores per inch (PPI) [24].

Models exist that quantify particle deposition into (or, alternatively penetration through) foam samplers based on a number of modifiable parameters, including flow rate, foam thickness and fiber diameter [24,25,26]. Of particular interest is a model developed specifically for foam used as an air sampling medium [24], described by the following function:

(6) $$\ln\left(P\right)=-\frac{t}{d_{f}}\left(40.7St^{1.90}+38.9Ng^{0.880}+84.36Pe^{-0.747}\right)$$

where *P* is the penetration through the foam, *t* is the foam thickness, and *d_f_* is the foam equivalent fiber diameter. *St* is the Stokes number, *Ng* is the gravitational settling number and *Pe* is the Peclet number, which taken together describe how a particle behaves in air. They are determined by the following equations:

(7) $$St=\frac{\rho_{0}{d_{ae}}^{2}UC_{c}}{18\eta d_{f}}$$

(8) $$Ng=\frac{\rho_{0}{d_{ae}}^{2}gC_{c}}{18\eta U}$$

(9) $$Pe=\frac{3\pi \eta d_{th}d_{f}U}{kTC_{c}}$$

where *ρ*_0_ is the density of water, *d_ae_* is the particle aerodynamic diameter, *U* is the air velocity through the foam, *C_c_* is the Cunningham slip correction factor, *η* is the viscosity of air, *g* is the acceleration due to gravity, *d_th_* is the particle thermodynamic equivalent diameter, *k* is Boltzmann’s constant, and *T* is temperature (Kelvin). It should be noted that the primary difference between this model and a previous one developed by Kenny *et al.* [26] is the addition of the Peclet number, which describes the tendency of a particle to move via diffusion.

**Figure 4:**
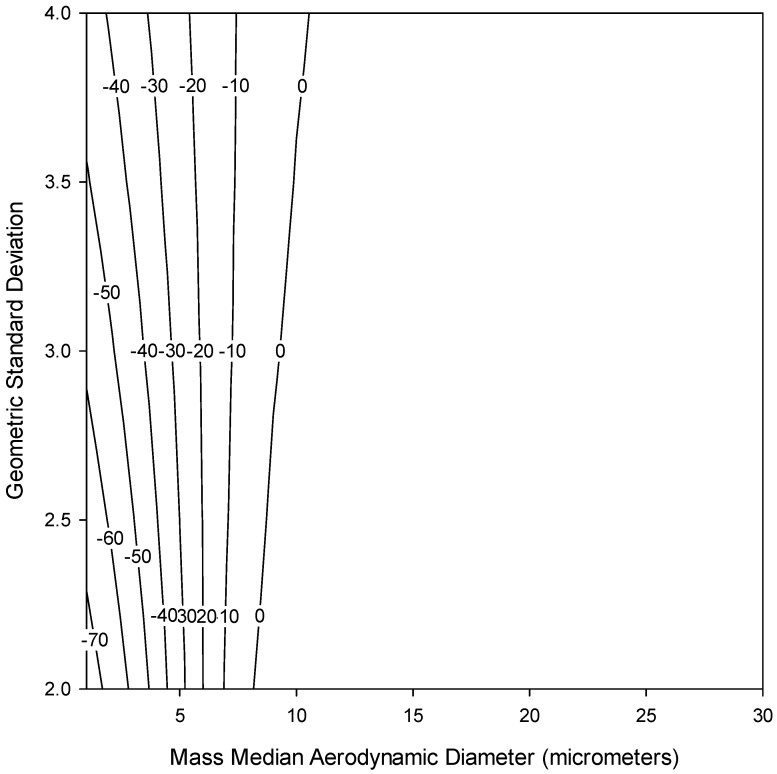
Estimated sampling bias (%) for the modeled IOM Multidust foam insert (Equation (6)) relative to the normalized extrathoracic (ET) deposition convention (Equation (4)) for a range of particle size distributions (mass median aerodynamic diameters up to 30 µm and geometric standard deviations between 2 and 4).

Since foam deposition, not penetration, is of interest to this study, by taking 1–(P) from Equation (6), we can then estimate the deposition of particles in different types of foam. The currently available IOM sampler foam was modeled for particles in the aerodynamic particle size region to assess the expected deposition (collection) efficiency of that insert. For a proper comparison, the modelled efficiency for the IOM foam was multiplied by inhalability, as given by Equation (5), with the assumption that the IOM inlet accurately selects for the inhalable aerosol fraction. Figure 3 shows this modelled efficiency (dotted line) compared to the normalized ET deposition convention (solid line). Some similarities are apparent, especially for large particles, although potentially important differences exist for particle sizes less than 10 µm. This is supported by a bias map, shown in Figure 4, which compares the normalized ET deposition convention with the modeled IOM foam (plotted together in Figure 3) for a range of particle size distributions (mass median aerodynamic diameters up to 30 µm with geometric standard deviations between 2 and 4). The clustering of bias lines at the smaller particle sizes implies that, as expected, the major differences between the modeled foam and the convention are in this lower size range. Overall, however, the similarities are strong enough that laboratory testing of this foam for comparison to the extrathoracic deposition curve was warranted. An additional consideration was that, in order to adequately plan for the potential adoption of a new sampling device, thought should be given at this stage to the existing technology and how it might be modified to suit this new purpose.

Therefore, due to its ability to simultaneously separate out the inhalable aerosol fraction and collect larger particles, the particle deposition efficiency of the IOM sampler foam was evaluated in this study as a measure for human extrathoracic deposition of inhaled particles. This would provide a novel method for extrathoracic deposition sampling using existing, commercially available technology.

### 2.2. Wind Tunnel

A low speed wind tunnel was used to represent typical indoor workplace wind speeds. Approximately 85% of indoor occupational wind speeds are 0.3 m/s or less [27]. The wind tunnel was designed to assess personal aerosol samplers in wind speeds of 0.1–0.5 m/s with minimal turbulence and uniform particle distribution [28]. The wind speed used during this study was 0.2 m/s. Wind speeds were confirmed using a micromanometer to assess pressure drop across the entrance filters as described in Schmees *et al.* [28]. These filters were changed periodically to prevent changes in pressure drop due to particle buildup. Samplers were attached to a life-size mannequin torso (Measurement Technology Northwest, Seattle, WA, USA) rotating reciprocally through 360° at two rotations per minute. The heating and breathing features outlined in Schmees *et al.* [28] were not used.

### 2.3. Aerosol Characterization

Brown fused aluminum oxide particles were used as the test aerosol (Duralum, Washington Mills, Niagara Falls, NY, USA). Five different particle sizes were used with mass median aerodynamic diameter (MMAD): 4.9 μm (Geometric Standard Deviation (GSD) = 1.73), 9.5 μm (1.32), 12.8 μm (1.47), 32.7 μm (1.71), and 44.3 μm (1.59). The particle sizes and GSDs were previously obtained using a modified Marple cascade impactor (see Schmees *et al.*, 2008) for sizes 9.5 µm (grit size = F1200), 12.8 µm (F800), 32.7 µm (F500) and 44.3 µm (F400). Particle size 4.9 µm was ground down from the 9.5 µm particle by the manufacturer for this study, so size was determined separately using a portable aerosol spectrometer (PAS) (GRIMM Technologies model 1.109, Douglasville, GA, USA) (MMAD = 4.9 µm; GSD = 1.73) and confirmed with an Aerodynamic Particle Sizer (APS Model 3321, TSI, Inc., Shoreview, MN, USA) (MMAD = 4.99 µm; GSD = 1.96).

### 2.4. Aerosol Generation

During each sampling event, only one particle size was introduced into the wind tunnel. Each particle size was used for two tests, in a randomly chosen order, resulting in ten sampling events. Each sampling event lasted for a period of 45 min. After each sampling event, the wind tunnel, mannequin and aerosol generator were cleaned with a high-efficiency particulate air (HEPA) vacuum to prevent cross-contamination of particle sizes.

Particles were aerosolized using a mechanical dust generator (SAG 410, Topas Gmbh, Dresden, Germany). The generator was connected via tubing to a dual-tracking particle dispersion system in the upstream mixing chamber. The uniformity of the particle dispersion system is described in Schmees *et al.* (2008). Temperature and relative humidity were documented. Two reference samples were collected for each sampling event using an isokinetic sampler located 0.75 m upstream of the mannequin. One additional isokinetic sampler was used as a blank. In addition to gravimetric analysis of the 25-mm glass fiber filter (1.0 µm pore size) from the isokinetic sampler, the internal wall deposits were collected using a cotton wipe soaked in isopropyl alcohol. After collection of the wall deposits, the cotton was desiccated and then analyzed gravimetrically.

### 2.5. Sampling Methods

Conductive plastic IOM samplers were used with stainless steel cassette assemblies. The IOM samplers were equipped with a 25-mm, 1.0 μm pore size, glass fiber filter and polyurethane foam insert (IOM MultiDust respirable foam disc, SKC Inc., Eighty-four, PA, USA). The stability of the foam was analyzed before beginning sampling to ensure its suitability for gravimetric analysis. In this analysis, foams were exposed to indoor ambient conditions over 10 days. Gravimetric analysis was performed each day using a semi-microbalance, readable to 0.01 mg (Sartorius model MSA225S100DI, Goettingen, Germany). Temperature and relative humidity were also documented. No significant gain or loss of mass was noted.

**Figure 5:**
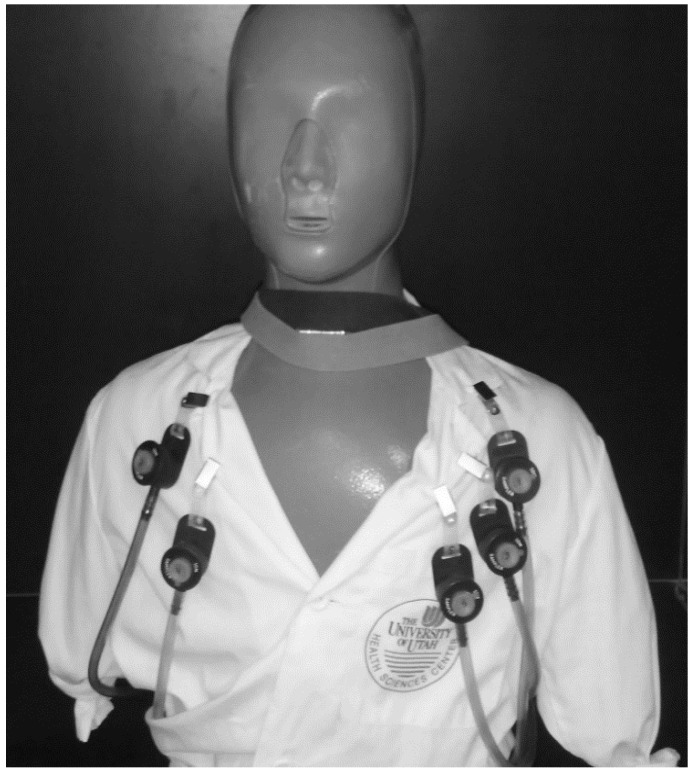
IOM sampler setup on a rotating mannequin in a low speed wind tunnel.

For each sampling event, five IOM samplers were placed on the anterior surface of the mannequin, conforming to standard occupational hygiene sampling procedures, with the mannequin located inside the wind tunnel, as shown in Figure 5. Two additional IOM samplers were used as blanks. Prior to the sampling event, each IOM sampler was connected to a pump, which was set to operate at 2 L/min using a BIOS DryCal primary flow meter (Bios International Corporation, Butler, NJ, USA). Pump flow rates were measured again after sampling to confirm constant airflow rate. Samples were excluded if they exceeded ±5% change in air flow. Gravimetric analysis was performed using the semi-microbalance mentioned previously. The IOM foam, filter and fully-assembled stainless steel IOM cassette were analyzed individually. The outer surface of the fully assembled cassettes were wiped prior to weighing to remove deposits on the outside of the inlet, which are not considered part of the sampled mass.

### 2.6. Data Analysis

To generate all bias maps, particle size distributions with MMAD between 1 and 50 µm and GSD between 2 and 4 (*n* = 250 distributions) were used to represent typical polydisperse aerosol found in workplaces. Data analysis was performed using Microsoft Excel and SigmaPlot 12.0 (Systat Software, Inc., San Jose, CA, USA).

## 3. Results

The results for the foam insert sampling efficiencies are shown in Table 1. Temperatures ranged from 24 °C to 26 °C in the gravimetric laboratory and 22–23 °C at the wind tunnel. Relative humidity was a constant 2% in the gravimetric laboratory and ranged from 2% to 23% (mean = 15.7%) at the wind tunnel. Although each particle size was tested multiple times in different experiments, the foam insert collection efficiencies from all samplers were combined within each particle size. It should be noted that the data shown in Table 1 only reflect half of the 4.9 µm samples. The isokinetic pump flow rate during the second 4.9 µm sampling event did not meet the a priori acceptance rate (±5%). However, despite the pre- and post-sampling pump flow rate changes of 8.62% and 8.80%, the mean inhalability-normalized foam collection efficiency (0.74, standard deviation (SD) = 0.01) was similar to the first 4.9 µm sampling event (0.68, SD = 0.01), as shown in Table 1. Ultimately, due to concerns with the isokinetic sampling pump operation during this experiment, these foam efficiency data were excluded from subsequent analysis.

Discrepancies with the isokinetic reference samplers are also evident in the mean collection efficiency results for the IOM inlet as a measure of the inhalable aerosol fraction, also shown in Table 1. In that case, the IOM sampler did not perform quite as expected compared to the isokinetic reference concentration. It is possible that wiping of the isokinetic reference sampler inlet is not as effective at collecting the wall deposits as rinsing the inlet; this concern is being investigated. However, for estimating the foam collection efficiency, all results are normalized to the inhalable fraction, which renders those isokinetic sampler results unnecessary. Therefore, this concern does not affect our conclusions.

**Figure 6:**
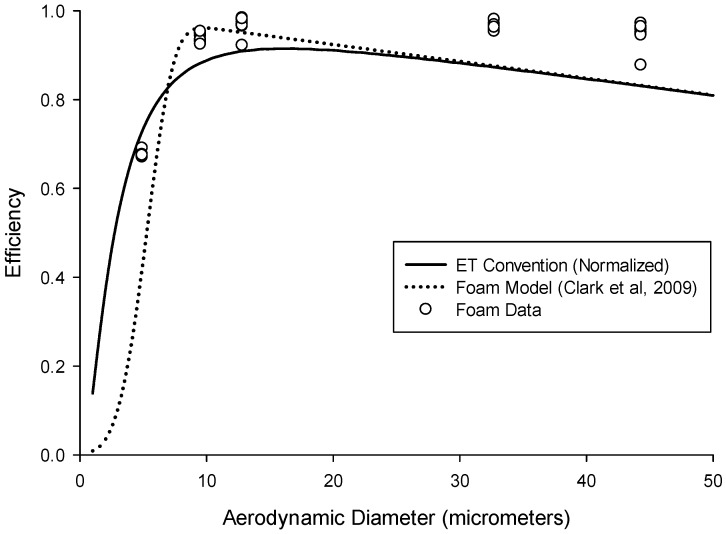
Foam insert sampling efficiency (circles, *n* = 45, with many circles overlapping due to tight reproducibility) compared to the normalized extrathoracic (ET) deposition sampling convention (solid line). The dotted line is the foam deposition efficiency as modeled by Clark *et al.* [24].

Figure 6 shows the foam efficiency data alongside both the ET convention and the modeled foam prediction. Overall, the foam efficiency data match well with that convention, with some tendency to underestimate for small particle sizes and overestimate for large particle sizes. The lack of data for particles below 4.9 µm, for which the ET convention rapidly changes, precludes the generation of usable bias maps to provide a quantitative comparison of the empirical data to the target specification. However, for the particle sizes tested, the data are consistent with both the ET convention and the foam model prediction.

Altogether, the data indicate that utilizing the existing IOM inhalable aerosol sampler with a PUF insert reasonably approximated the normalized extrathoracic deposition convention for particles larger than 4.9 µm. Repeatability, both within and between experimental runs, was good, as evidenced by standard deviations ≤0.03 for all particle sizes (see Table 1). This specific PUF sampling media proved easy to use with acceptable weight stability for gravimetric analysis. Therefore, the use of this foam for measuring extrathoracic particle deposition shows considerable promise, and these results demonstrate that this concept merits additional study, e.g., testing of additional particle sizes and modifications to adjust the sampling efficiency to better match that deposition convention.

## 4. Discussion

The purpose of this laboratory study was to determine if the IOM sampler with a foam insert could be used as an accurate measure for human extrathoracic particle deposition. The foam model prediction and the experimental data provide a “proof of concept” that the IOM sampler with foam insert may be effective at measuring the human extrathoracic deposition fraction (ISO ET convention). These results document the feasibility of this approach, although the foam insert performance for particles between 1 µm and 5 µm, for which the sampling efficiency rapidly changes, is still unknown. Further investigation using this existing sampler for this new purpose is therefore of continued interest.

One limitation of this sampler design is that it is based only on the aerodynamic size range of particles. In practice, there may be exposures that include particles in the thermodynamic size range (<0.5 µm) as well. For gravimetric analysis, the contribution of these smaller particles might not be significant. However, for other analytical techniques (e.g., metals analysis), this limitation should be kept in mind.

A minor limitation of this study was the mechanism in which the 4.9 µm particle was measured. Since this particle size was newly acquired, the MMAD and GSD were measured using a GRIMM model 1.109 Portable Aerosol Spectrometer (PAS) (GRIMM Technologies, Douglasville, GA, USA) and an Aerodynamic Particle Sizer (APS) Model 3321 (TSI, Inc., Shoreview, MN, USA). All other particles were sized in a previous study using a modified Marple personal cascade impactor (Model 290, from SKC Inc., Eighty Four, PA, USA) [28]. Other studies have noted that the GRIMM 1.109 PAS produced similar results with respect to sizing of particles as the APS [29], and similar results were found in this study. The 4.9 µm MMAD with narrow GSD reported by the GRIMM PAS was expected due to the fact that it was produced through grinding down the 9.5 µm particle size (*i.e.*, the company was instructed to provide a size one half as large).

Finally, PUF material can be quite hygroscopic, and so its use for gravimetric analysis has been previously questioned [12]. Our initial examination of the weight stability of this foam (with relative humidity (RH) typically between 10% and 30%) showed no significant gain or loss of mass over a 10 days period. However, during the wind tunnel experiments, RH in the gravimetric laboratory averaged 2% while the wind tunnel ambient RH averaged 15.7%. Such low RH could potentially provide skewed results in this case, whereby moisture uptake by the PUF would typically be quite low. Further testing at much higher RH is therefore recommended. It should be noted, however, that the foam used for this sampler is sold as part of the IOM Multidust sampler, which requires weighing the foam with the rest of the sampler to provide an estimate of the inhalable fraction. It is unclear if the manufacturer has treated this foam to enable gravimetric analysis, but it does point to the fact that the type of PUF utilized for sampling will be important to consider. Overall, despite less than optimal laboratory conditions, the results from this study were repeatable both within and between different sampling events.

### Future Work

**Figure 7:**
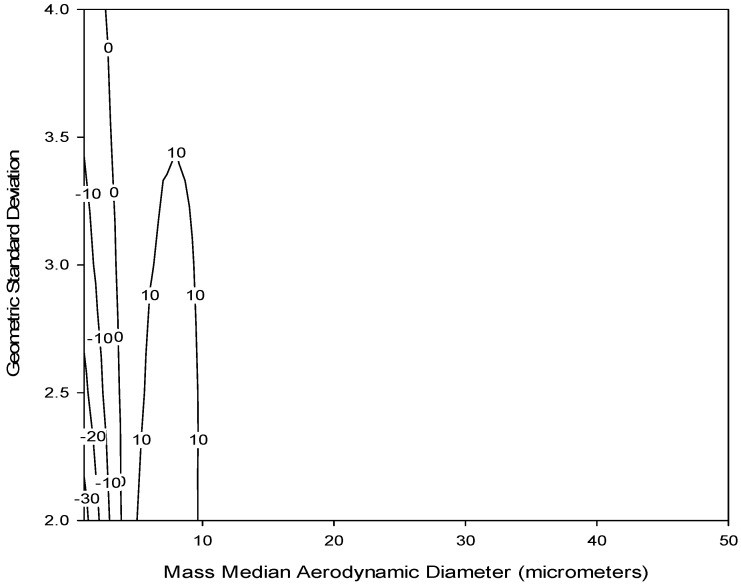
Estimated sampling bias (%) for the modeled foam insert operated at 5 L/min (using Equation (6)) relative to the normalized extrathoracic (ET) deposition convention (Equation (4)) for a range of particle size distributions (MMAD up to 50 µm and GSD between 2 and 4).

Utilizing the model in Equation (6), it is possible to identify some potential parameters that might be modified to improve this sampler's performance relative to the convention. This includes possibly increasing the sampling flow rate, reducing the foam thickness, or using foam with smaller fiber diameter. In the simplest case, it appears that by increasing the sampling flow rate from 2 L/min to 5 L/min, a better agreement between this foam deposition efficiency and that criterion may be achievable. Figure 7 shows a bias map comparing the foam insert modeled at a 5 L/min flow rate compared to the extrathoracic deposition convention for a range of particle size distributions (MMAD up to 50 µm with GSD between 2 and 4). Again, the bias lines are clustered at the smaller particle sizes as was seen in Figure 4, but in this case the bias is substantially reduced for those smaller sizes. A higher flow rate also has the added benefit of improving the sampler's ability to detect substances present at low concentrations, which is especially important for substances that have increasingly lower occupational exposure limits. However, more work will need to be done to ensure that the sampler maintains its sampling efficiency for inhalable particles in order to use the higher flow rate. In that regard, other studies have indicated that the IOM can be operated at higher flow rates with only minimal effects on sampling efficiency, as reported for both computational fluid dynamics [30] and under laboratory conditions [31]. If the use of entirely different foam was desired, it could also be valuable to investigate simultaneous changes to multiple parameters, e.g., foam thickness and fiber diameter, to achieve better agreement.

Relatedly, non-gravimetric, elemental analysis of airborne contaminants (*i.e.*, hazardous metals) is also of interest to both demonstrate generalizability of this sampler, as well as to document suitability for consideration of use in a wider array of workplaces. Recent methods for digestion and analysis of PUF using inductively-coupled plasma-mass spectroscopy (ICP-MS) [32] could be useful in this respect. An additional consideration for the development of an air sampler, especially one that should be deployable to field sites, is the ease of use and robustness (*i.e.*, the ability to tolerate heavy activity and transport with minimal effect) of the design. To that end, field testing of this device should be carried out for occupational environments with extrathoracic hazards (e.g., wood dust).

## 5. Conclusions

The concept of using a foam insert to match a particle deposition sampling convention has been explored in this study. The experimental data support that the IOM sampler with foam insert may be a viable method to measure human extrathoracic deposition with minimal bias, but further modification and testing are warranted.

## Figures and Tables

**Table 1:** IOM foam insert efficiency results.

Mass Median Aerodynamic Diameter (µm)	Geometric Standard Deviation	No. of Samples (*n*)	Mean Inhalable Collection Efficiency ^c^ (Standard Deviation)	Mean Foam Collection Efficiency ^d^ (Standard Deviation)	Normalized Foam Collection Efficiency ^e^ (Standard Deviation)	Reference Extrathoracic Deposition Efficiency ^f^
4.9 ^a^	1.73 ^a^	5	0.79 (0.01)	0.53 (0.01)	**0.68 (0.01)**	0.73
9.5 ^b^	1.32 ^b^	10	0.59 (0.06)	0.56 (0.05)	**0.94 (0.01)**	0.88
12.8 ^b^	1.47 ^b^	10	0.89 (0.07)	0.86 (0.07)	**0.97 (0.02)**	0.96
32.7 ^b^	1.71 ^b^	10	0.83 (0.21)	0.80 (0.20)	**0.97 (0.01)**	0.87
44.3 ^b^	1.59 ^b^	10	0.78 (0.16)	0.74 (0.16)	**0.95 (0.03)**	0.83

^a^ Determined using a Portable Aerosol Spectrometer (GRIMM Technologies model 1.109, Douglasville, GA, USA); ^b^ Obtained from Schmees *et al.* (2008) [28]; ^c^ Calculated as inhalable mass concentration measured by the IOM sampler divided by the reference concentration; ^d^ Calculated as mass concentration measured by the foam divided by the reference concentration; ^e^ Normalized foam efficiency is the foam collection efficiency divided by the inhalable collection efficiency; ^f^ Calculated using Equation (4).

## Abbreviations

The following abbreviations are used in this manuscript:

ICP-MS: Inductively-Coupled Plasma – Mass Spectroscopy
ISO: International Organization for Standardization
ET: Extrathoracic
ET_1_: Anterior Nasal Passage
ET_2_: Posterior Nasal and Oral Passages
ICRP: International Committee for Radiological Protection
BB and bb: Tracheobronchial
PUF: Polyurethane Foam
PPI: Pores Per Inch
MMAD: Mass Median Aerodynamic Diameter
GSD: Geometric Standard Deviation
PAS: Portable Aerosol Spectrometer
APS: Aerodynamic Particle Sizer
HEPA: High-Efficiency Particulate Air
SD: Standard Deviation
RH: Relative Humidity
